# Supplementary material for: Altruistic feeding and cell-cell signaling during bacterial differentiation actively enhance phenotypic heterogeneity
Source: Sci Adv. 2024 Oct 18;10(42):eadq0791. doi: 10.1126/sciadv.adq0791 (PMC11488536; doi:10.1126/sciadv.adq0791)
Supplement: Supplementary file 1 — Figs. S1 to S4 Table S1 Uncropped immunoblots [file sciadv.adq0791_sm.pdf]

Science Advances
AAAS

Supplementary Materials for

Altruistic feeding and cell-cell signaling during bacterial differentiation actively enhance phenotypic heterogeneity

Taylor B. Updegrove *et al.*

Corresponding author: Kumaran S. Ramamurthi, ramamurthiks@mail.nih.gov

Sci. Adv. **10**, eadq0791 (2024)
DOI: 10.1126/sciadv.adq0791

This PDF file includes:

Figs. S1 to S4
Table S1
Uncropped immunoblots

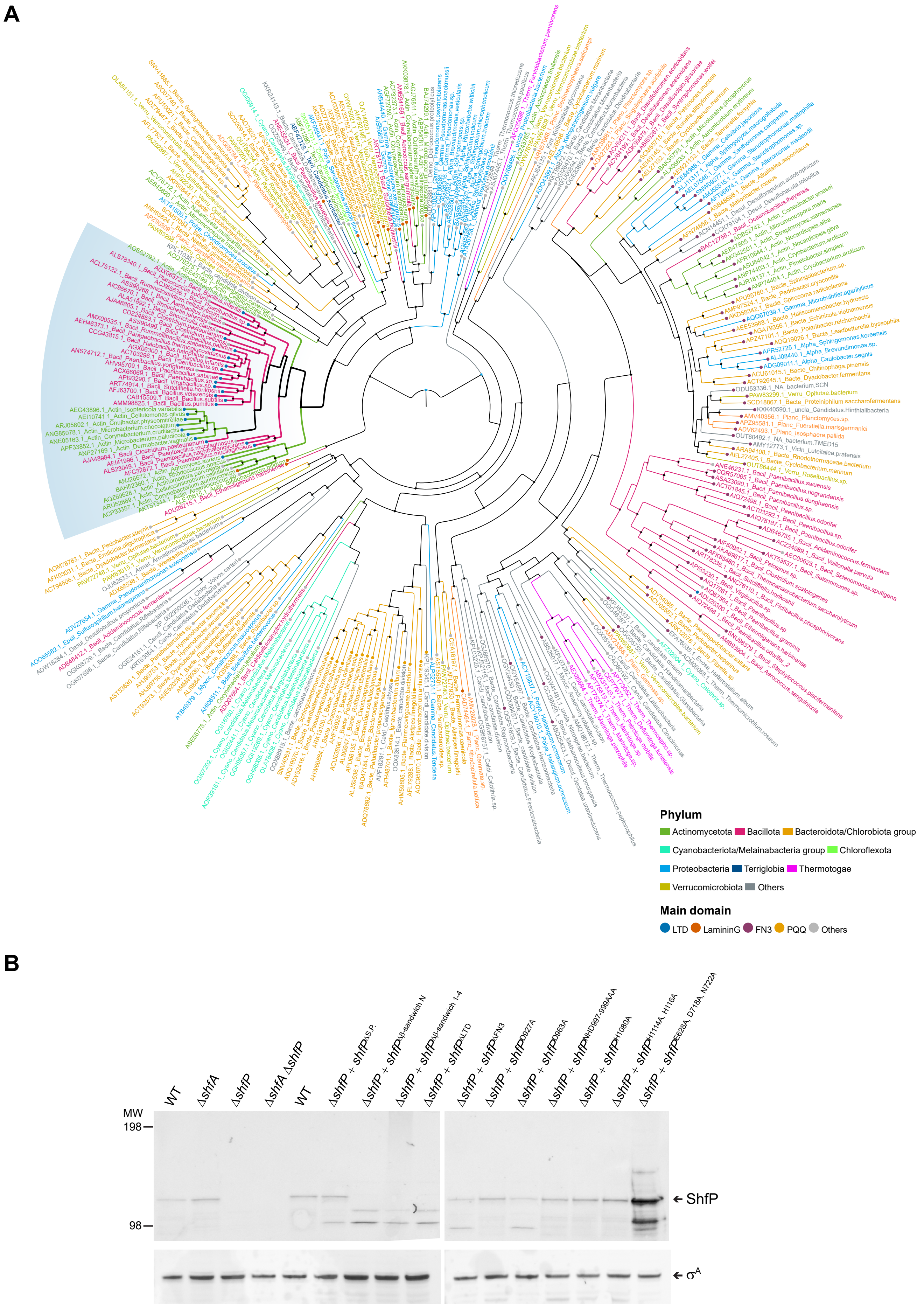

A
Phylum
Actinomycetota
Bacillota
Bacteroidota/Chlorobiota group
Cyanobacteriota/Melainabacteria group
Chloroflexota
Proteobacteria
Terriglobia
Thermotogae
Verrucomicrobiota
Others
Main domain
LTD
LamininG
FN3
PQQ
Others
B
WT
ΔshfA
ΔshfP
ΔshfA ΔshfP
WT
ΔshfP + shfP ΔS.P.
ΔshfP + shfP Δβ-sandwich N
ΔshfP + shfP Δβ-sandwich 1-4
ΔshfP + shfP ΔLTD
ΔshfP + shfP ΔFN3
ΔshfP + shfP D927A
ΔshfP + shfP D963A
ΔshfP + shfP NHD997-999AAA
ΔshfP + shfP H1080A
ΔshfP + shfP H1114A, H116A
ΔshfP + shfP E628A, D718A, N722A
MW
198
98
ShfP
σA

Fig. S1. Phylogenetic analysis of ShfP and calcineurin-like proteins. (A) A phylogenetic tree of a representative set of extracellular calcineurin-like domains from the homologs of the ShfP family is shown. The name of each member is shown with its accession number, phylum, and organism name. (B) Immunoblot against ShfP (top panel) for the following strains: WT (lane 1); Δ*shfA* mutant (lane 2); Δ*shfP* mutant (lane 3); Δ*shfA* Δ*shfP* mutant (lane 4); Δ*shfA* Δ*shfP* *amyE*::*shfP* (lane 5); Δ*shfA* Δ*shfP* *amyE*::*shfP*$^{\Delta 2\text{-}28}$ (lane 6); Δ*shfA* Δ*shfP* *amyE*::*shfP*$^{\Delta 27\text{-}139}$ (lane 7); Δ*shfA* Δ*shfP* *amyE*::*shfP*$^{\Delta 293\text{-}732}$ (lane 8); Δ*shfA* Δ*shfP* *amyE*::*shfP*$^{\Delta 140\text{-}280}$ (lane 9); Δ*shfA* Δ*shfP* *amyE*::*shfP*$^{\Delta 1154\text{-}1289}$ (lane 10); *f* Δ*shfP* *amyE*::*shfP*$^{\mathrm{D927A}}$ (lane 11); Δ*shfA* Δ*shfP* *amyE*::*shfP*$^{\mathrm{D963A}}$ (lane 12); Δ*shfA* Δ*shfP* *amyE*::*shfP*$^{\mathrm{NHD997\text{-}999AAA}}$ (lane 13); Δ*shfA* Δ*shfP* *amyE*::*shfP*$^{\mathrm{H1080A}}$ (lane 14); Δ*shfA* Δ*shfP* *amyE*::*shfP*$^{\mathrm{H114A,\ H116A}}$ (lane 15); Δ*shfA* Δ*shfP* *amyE*::*shfP*$^{\mathrm{E628A,\ D718A,\ N722A}}$ (lane 16). Anti-SigA used as a loading control (bottom panel). Strains: PY79; CW202; TD517; TD507; CC2; CC19; CC131; CC20; CC138; CC14; CC15; CC16; CC17; CC18; CC166.

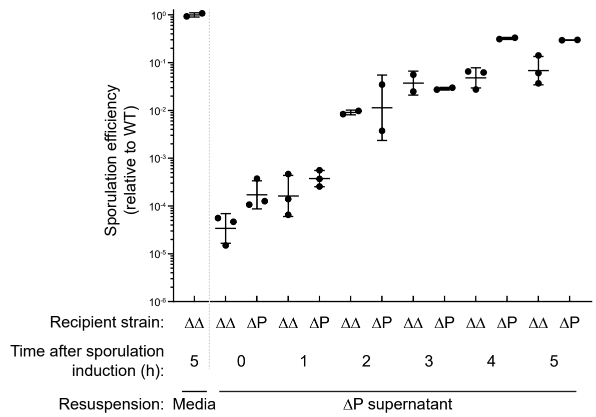

Fig. S2. Extracellular glycerol inhibits sporulation. Cell-free supernatant derived from the Δ*shfP* mutant strain shows no sporulation suppression activity. Sporulation efficiency of the Δ*shfA* Δ*shfP* (TD507) and Δ*shfP* (TD517) mutant recipient strains cultured for the indicated times in synchronous sporulation media prior to the addition of cell-free supernatants derived from the Δ*shfP* mutant strain. Sporulation efficiencies are reported as normalized values to that of the recipient strain cultured in the absence of supernatant. Mean and standard deviation of two or more independent cultures for each condition are shown.

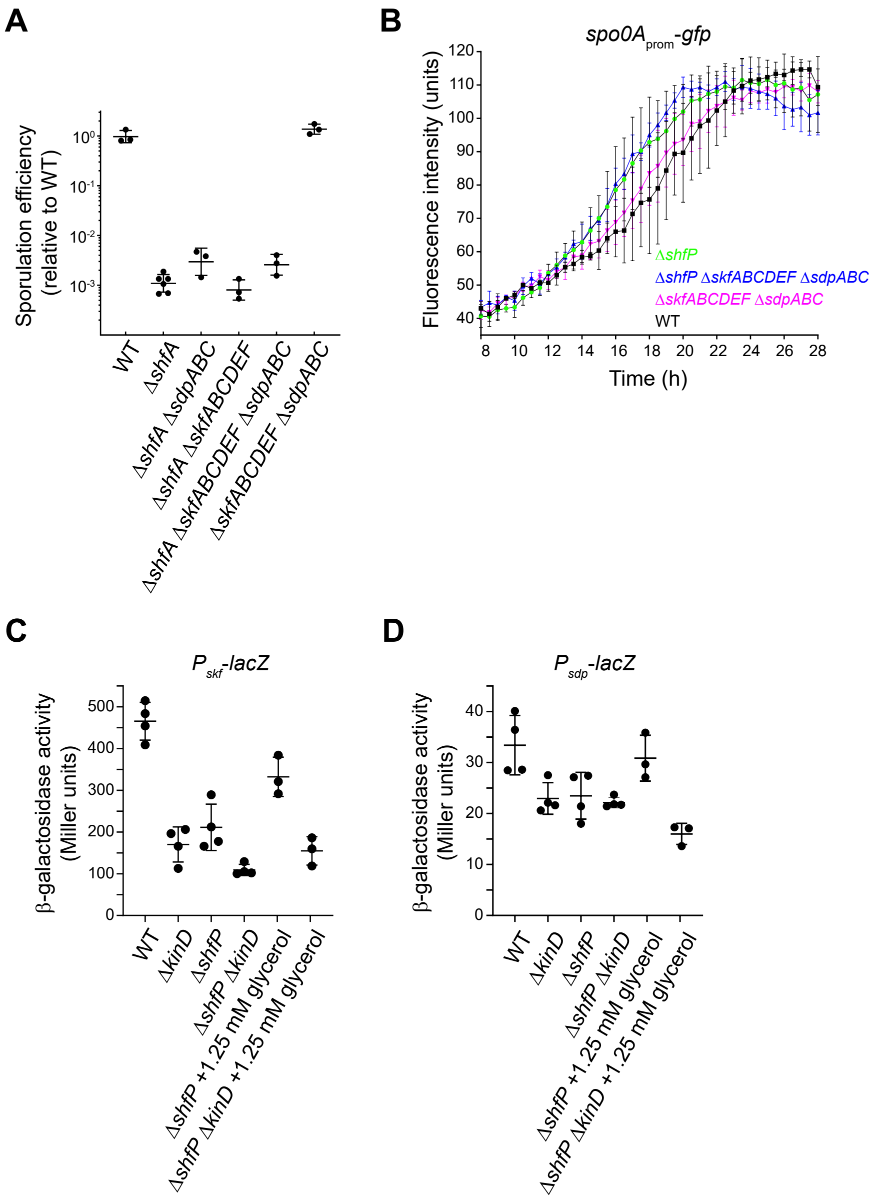

Fig. S3. The ShfA-ShfP pathway operates independently of the cannibalism pathway. (A) Sporulation efficiencies, as measured by resistance to 80°C for 20 min relative to WT, of indicated *B. subtilis* strains. Bars represent mean values; data points represent sporulation efficiencies from an independent culture. Strains: PY79; CW202; EG523; TU133; TU134; TU135 (B) Fluorescence intensity of indicated strains harboring the P_{spoIIE}-*gfp* reporter induced to sporulate by gradual nutrient deprivation. Strains: TU09; TU11; TU121; TU123. (C-D) Activation of the KinD-influenced promoters (C) P_{skf} or (D) P_{sdp} in the indicated strains, or in Δ*shfP* or Δ*shfP* Δ*kinD* cells in the presence of 1.25 mM glycerol in the medium. Strains: TU128; TU130; TU132; TU139; TU127; TU129; TU131; TU138.

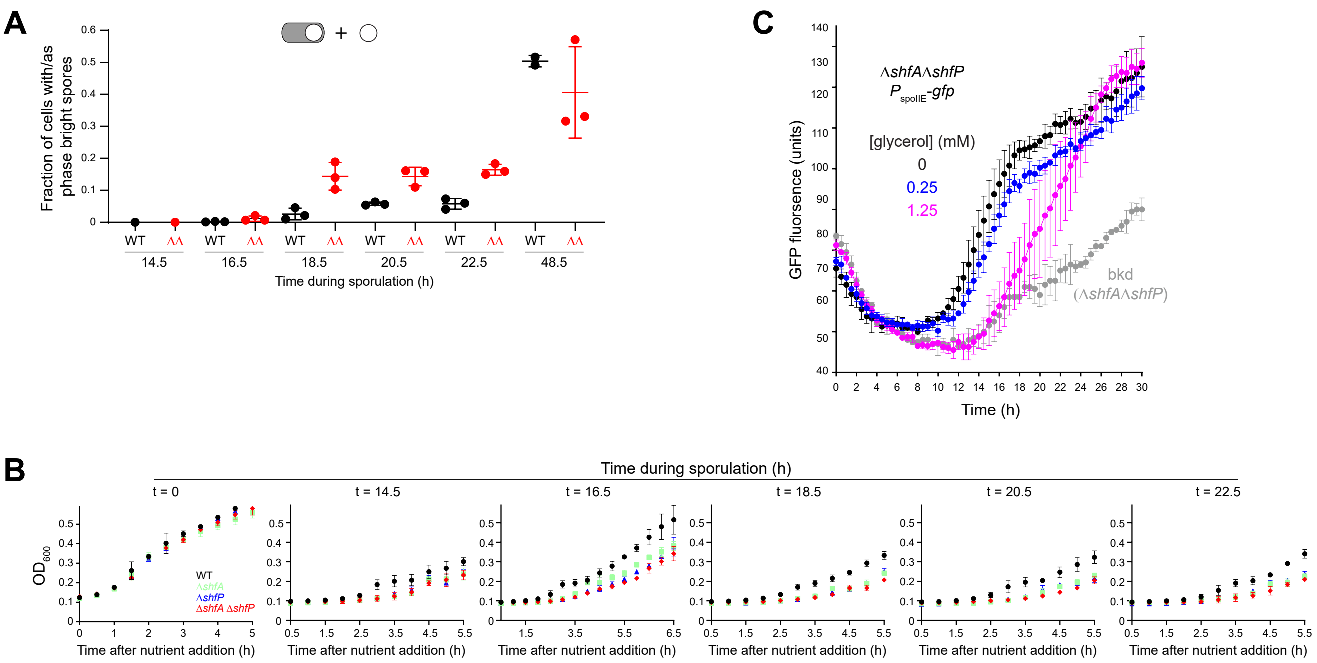

Fig. S4. Glycerol secretion delays formation of DIC-bright cells. (A) Fraction of (black) WT (MF277) or (red) Δ*shfA* Δ*shfP* (CC219) cells forming DIC-bright forespore or mature spore structures (as shown in the cartoon depictions above) at the indicated time points. Bars represent mean; errors: S.D.; data points represent a measure from an independent experiment. (B) WT cells respond faster to nutrient replenishment than Δ*shfA* Δ*shfP* mutant cells when grown under asynchronous sporulation conditions. WT (MF277), Δ*shfA*(CC218), Δ*shfP* (CC220), and Δ*shfA* Δ*shfP* (CC219) mutant strains were grown in asynchronous sporulation media. At the indicated time points, 10% of each culture was back diluted in fresh nutrient rich LB media and the OD600 was measured over time. Mean and standard deviation of three independent cultures are shown. The initial doubling time for each strain at the indicated time points of subculturing were used for Fig 5B. (C) The addition of glycerol to sporulation media causes a delay in entry into sporulation. The Δ*shfA* Δ*shfP* P_{spoIIE}-*gfp* (CC219) reporter strain and the non-gfp fusion strain Δ*shfA* Δ*shfP* (TD507) were grown in synchronous sporulation media with the indicated amount of glycerol added. GFP fluorescence was recorded over time (hours). Mean and standard deviation of four or more independent cultures for each strain and condition are shown.

Table S1. *Bacillus subtilis* strains used in this study.

Strains		
Name	Genotype	Source
PY79	Prototrophic derivative of *B. subtilis* 168.	(*47*)
CW202	*ΔyabQ::tet*	This study
TD517	*ΔyvnB::erm*	This study
TD507	*ΔyabQ::tet ΔyvnB::erm*	This study
CC2	*ΔyabQ::tet, ΔyvnB::erm amyE::yvnB cat*	This study
CC14	*ΔyabQ::tet, ΔyvnB::erm, amyE::yvnB*^{D927A} *cat*	This study
CC15	*ΔyabQ::tet, ΔyvnB::erm, amyE::yvnB*^{D963A} *cat*	This study
CC16	*ΔyabQ::tet, ΔyvnB::erm, amyE::yvnB*$^{NHD997\text{-}999AAA}$ *cat*	This study
CC17	*ΔyabQ::tet, ΔyvnB::erm, amyE::yvnB*^{H1080A} *cat*	This study
CC18	*ΔyabQ::tet, ΔyvnB::erm, amyE::yvnB*$^{H1114A,\ H1116A}$ *cat*	This study
CC19	*ΔyabQ::tet, ΔyvnB::erm, amyE::yvnB*$^{\Delta 2\text{-}28}$ *cat*	This study
CC20	*ΔyabQ::tet, ΔyvnB::erm, amyE::yvnB*$^{\Delta 140\text{-}280}$ *cat*	This study
CC131	*ΔyabQ::tet, ΔyvnB::erm, amyE::yvnB*$^{\Delta 27\text{-}139}$ *cat*	This study
CC138	*ΔyabQ::tet, ΔyvnB::erm, amyE::yvnB*$^{\Delta 1154\text{-}1289}$ *cat*	This study
CC166	*ΔyabQ::tet, ΔyvnB::erm, amyE::yvnB*$^{E628A,\ D718A,\ N722A}$ *cat*	This study
CC232	*ΔyabQ::tet, ΔyvnB::erm, thrC::yabQ spec amyE::yvnB cat*	This study
CC175	*ΔyabQ::tet, amyE::yvnB*$^{1\text{-}26}$*-sfGFP-yvnB*$^{140\text{-}1289}$ *cat*	This study
CC179	*amyE::yvnB*$^{1\text{-}26}$*-sfGFP-yvnB*$^{140\text{-}1289}$ *cat*	This study
CC105	*ΔyabQ::tet, amyE::P*$_{yvnB}$*-GFP cat*	This study
CC188	*ΔsigF::kan, amyE:: P*$_{yvnB}$*-sfGFP cat*	This study
CC189	*ΔsigE::erm, amyE:: P*$_{yvnB}$*-sfGFP cat*	This study
CC190	*ΔsigG::kan, amyE:: P*$_{yvnB}$*-sfGFP cat*	This study
CC191	*ΔspoIVCA::erm, amyE:: P*$_{yvnB}$*-sfGFP cat*	This study
MF277	*amyE::P*$_{spoIIE}$*-GFP spec*	(*10*)
CC218	*amyE::P*$_{spoIIE}$*-GFP spec ΔyabQ::tet*	This study
CC219	*amyE::P*$_{spoIIE}$*-GFP spec ΔyabQ::tet, ΔyvnB::erm.*	This study
CC220	*amyE:: P*$_{spoIIE}$*-GFP spec, ΔyvnB::erm*	This study
TU36	*amyE::P*$_{spoIIQ}$*-yvnB cat ΔyvnB::erm*	This study
TU40	*amyE::P*$_{spoIIQ}$*-yvnB*^{H1080A} *cat ΔyvnB::erm*	This study
TU41	*amyE::P*$_{spoIIQ}$*-yvnB*^{D927A} *cat ΔyvnB::erm*	This study
CC154	*ΔyvnB::erm amyE:P*$_{spoVM}$*-yvnB cat*	This study
CC160	*ΔyvnB::erm amyE::P*$_{sspB}$*-yvnB cat*	This study
TU46	*amyE::P*$_{hyperspank}$*-spoVM-GFP spec sacA::P*$_{sspB}$*-yvnB*$^{1\text{-}440}$*-mCherry-yvnB*$^{441\text{-}1290}$ *cat*	This study
TU53	*ΔthrC::P*$_{hyperspank}$*-spoVM-mCherry erm ΔamyE::P*$_{hyperspank}$*-gfp-spoIVA spec*	This study
CC254	*yvnB::erm amyE::yvnB*$^{NHD997\text{-}999AAA}$ *cat*	This study
CC255	*yvnB::erm amyE::yvnB*^{H1080A} *cat*	This study
CC258	*yvnB::erm amyE::yvnB*^{D927A} *cat*	This study
CC134	*ΔyvnB::erm, amyE::yvnB cat*	This study
CC221	*ΔyabQ::tet, ΔyvnB::erm, thrC::Pyab-yabQ-spec*	This study
TU69	*amyE::P*$_{spoIIE}$*-gfp spec ΔkinD::erm*	This study
TU78	*amyE::P*$_{spoIIE}$*-gfp spec ΔglpF::kan ΔglpK::erm*	This study
TU80	*amyE::P*$_{spoIIE}$*-gfp spec::chlor ΔkinD::P*$_{hyperspank}$*-kinD spec ΔglpF::kan ΔglpK::erm*	This study
TU84	*amyE::P*$_{spoIIE}$*-gfp spec ΔglpF::kan ΔglpK::erm ΔkinD::tet*	This study
TU87	*amyE::P*$_{epsA}$*-lacZ cat*	This study
TU88	*amyE::P*$_{epsA}$*-lacZ cat ΔkinD::tet*	This study
TU89	*amyE::P*$_{epsA}$*-lacZ-cat ΔyvnB::erm*	This study
TU92	*amyE::P*$_{epsA}$*-lacZ cat ΔkinD::tet ΔyvnB::erm*	This study
EG523	*ΔskfABCDEF::tet ΔsdpABC::spec*	(*14*)
TU133	*ΔskfABCDEF::tet ΔsdpABC::spec ΔyabQ::kan*	This study
TU134	*ΔskfABCDEF::tet ΔyabQ::kan*	This study
TU135	*ΔsdpABC::erm ΔyabQ::kan*	This study
TU09	*amyE::P*$_{spoIIE}$*-gfp spec*	

TU11	*amyE::P$_{spoIIE}$-gfp spec ΔyvnB::erm*	This study
TU121	*amyE::P$_{spoIIE}$-gfp spec ΔskfABCDEF::tet ΔsdpABC::erm*	This study
TU123	*amyE::P$_{spoIIE}$-gfp spec ΔskfABCDEF::tet ΔsdpABC::erm ΔyvnB::kan*	This study
TU128	*amyE::P$_{skf}$-lacZ cat*	This study
TU130	*amyE::P$_{skf}$-lacZ cat ΔkinD::erm*	This study
TU132	*amyE::P$_{skf}$ -lacZ cat ΔyvnB::kan*	This study
TU139	*amyE::P$_{skf}$ -lacZ cat ΔkinD::erm ΔyvnB::kan*	This study
TU127	*amyE::P$_{sdp}$-lacZ cat*	This study
TU129	*amyE::P$_{sdp}$-lacZ cat ΔkinD::erm*	This study
TU131	*amyE::P$_{sdp}$-lacZ cat ΔyvnB::kan*	This study
TU138	*amyE::P$_{sdp}$-lacZ cat ΔkinD::erm ΔyvnB::kan*	This study
TU148	*ΔglpF::kan ΔglpK::erm*	This study

Anti-ShfP

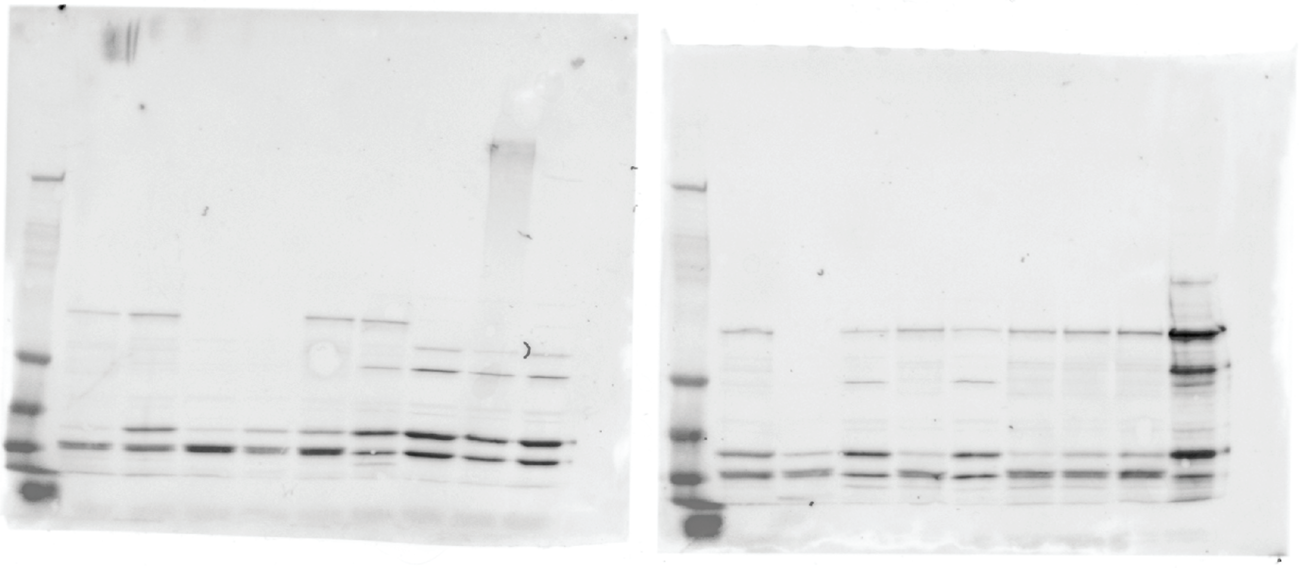

Anti-σ^A

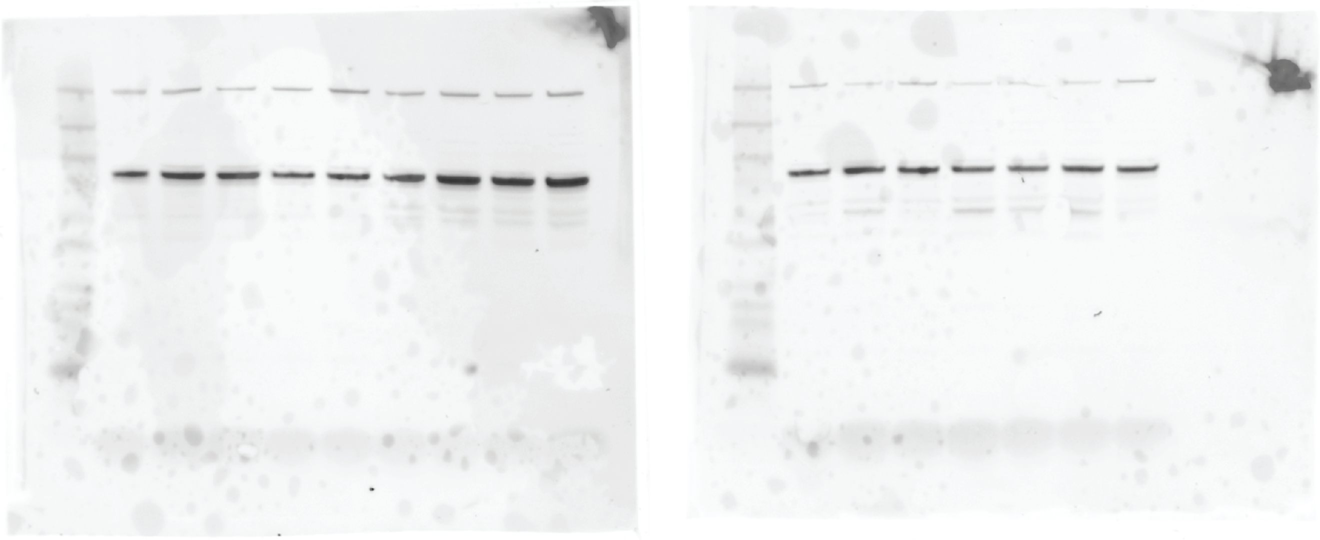

Uncropped immunoblot images for Fig. S1B. Top row: anti-ShfP (YvnB); bottom row: anti σ^A.
